# A PRCI-Based Individualized Risk Prediction Model for Potentially Inappropriate Medication in Older Outpatients

**DOI:** 10.3390/jcm15197675

**Published:** 2026-10-04

**Authors:** Zhaoyan Chen, Congjun Zhou, Yongfei Dong, Cairong Zhu

**Affiliations:** Department of Epidemiology and Health Statistics, West China School of Public Health and West China Fourth Hospital, Sichuan University, Chengdu 610041, China18349482477@163.com (C.Z.); dyongfei99@163.com (Y.D.)

**Keywords:** potentially inappropriate medications, older, risk, outpatient

## Abstract

**Objectives**: Potentially inappropriate medication (PIM) use remains highly prevalent among older outpatients, yet individualized risk prediction tools are limited. This study aimed to develop and validate a PIM risk prediction model incorporating a novel disease-weighted index. **Methods**: A multicenter cross-sectional study was conducted using 131,894 outpatient prescriptions from 59 medical institutions in China. Diagnoses were mapped to ICD-10 codes. A PIM Risk-Related Comorbidity Index (PRCI) was developed using multivariable logistic regression and coefficient-weighted scoring. A prediction model was constructed and internally validated using a 6:4 split-sample approach. Discrimination, calibration, and clinical utility were assessed using ROC curves, calibration plots, and decision curve analysis. **Results**: The proportion of prescriptions containing at least one PIM was 29.0%. Polypharmacy showed a strong dose–response relationship with PIM risk. The PRCI was independently associated with PIM (OR = 1.07 per unit increase). The final model demonstrated good discrimination (AUC = 0.775 in training and 0.773 in validation), satisfactory calibration, and favorable clinical utility. A cutoff probability of 0.304 stratified prescriptions into high- and low-risk groups. **Conclusions**: The PRCI-based model provides an individualized and clinically applicable tool for identifying older outpatient prescriptions at high risk of containing PIMs, supporting proactive medication safety management.

## 1. Introduction

Population aging has emerged as one of the most significant public health challenges worldwide. By the end of 2021, China had approximately 191 million individuals aged 65 years and older, accounting for 13.5% of the total population and nearly one-quarter of the global older population. Along with increased life expectancy, the prevalence of chronic diseases and multimorbidity has risen substantially among older adults. More than 75% of older Chinese adults live with at least one chronic condition, and many require long-term pharmacological treatment involving multiple medications [1]. Consequently, polypharmacy, commonly defined as the concurrent use of five or more medications, has become increasingly prevalent in geriatric populations and is now recognized as a major contributor to medication-related harm [2]. Older adults are particularly vulnerable to inappropriate prescribing because of age-related physiological changes affecting drug absorption, distribution, metabolism, and excretion. Potentially inappropriate medications (PIMs) refer to medications for which the potential risks outweigh the anticipated clinical benefits when safer or more effective alternatives are available [3]. Extensive evidence has demonstrated that PIM use is associated with numerous adverse outcomes, including falls, fractures, cognitive impairment, hospitalization, healthcare utilization, and mortality [4,5,6,7]. Beyond its impact on individual health, PIM-related harm imposes a substantial economic burden on healthcare systems worldwide.

Among all healthcare settings, older outpatients represent a particularly high-risk population. Unlike hospitalized patients whose medications are continuously monitored by multidisciplinary healthcare teams, outpatient medication management largely depends on self-administration or caregiver supervision. Long treatment durations, frequent prescription renewals, fragmented healthcare utilization, and limited professional monitoring collectively increase the likelihood of inappropriate medication use [8]. Previous studies have suggested that the adverse consequences associated with PIM exposure may be more pronounced among community-dwelling and outpatient older adults than among hospitalized patients [9,10]. Given that outpatient clinics serve as the primary source of chronic disease management and medication prescribing for older individuals in China, improving medication safety in this setting remains a public health priority.

To identify PIMs, several explicit screening tools have been developed and widely adopted, including the Beers Criteria, STOPP/START Criteria, and the Chinese Criteria for Potentially Inappropriate Medication Use in Older Adults [11,12,13]. These instruments provide valuable guidance for medication review and prescribing optimization. However, most existing tools function primarily as retrospective screening instruments that identify inappropriate medications after prescribing decisions have already occurred. Although effective for quality assessment, they offer limited support for prospective risk stratification and early intervention.

Recent studies have increasingly focused on identifying predictors of PIM exposure [14,15,16,17]. Nevertheless, several important limitations remain. First, most studies evaluate individual diseases independently or simply quantify multimorbidity using disease counts. Such approaches implicitly assume that all diseases contribute equally to PIM risk, which may not reflect real-world prescribing practices. Second, considerable heterogeneity exists in the magnitude and direction of associations between different chronic conditions and PIM exposure. Certain diseases may substantially increase the likelihood of inappropriate prescribing because of complex therapeutic regimens, whereas others may have minimal influence. Third, existing evidence has rarely been translated into practical clinical tools capable of quantifying the cumulative risk associated with an individual’s overall disease profile.

To address these limitations, we developed a novel PIM Risk-Related Comorbidity Index (PRCI), a disease-weighted scoring system designed to quantify the cumulative contribution of chronic disease patterns to PIM risk. Unlike conventional multimorbidity measures based solely on disease counts, the PRCI incorporates the relative effect size of each disease on PIM occurrence, thereby capturing the heterogeneous impact of different comorbidities. By transforming complex disease information into a single continuous score, the PRCI provides a practical and interpretable measure of patient-level vulnerability to inappropriate medication use.

Therefore, the present study aimed to develop and validate a PRCI-based individualized risk prediction model for PIM among older outpatients. Using a large multicenter outpatient dataset, we sought to identify independent predictors of PIM, construct a clinically applicable prediction model incorporating the PRCI, and evaluate its discrimination, calibration, and clinical utility. We hypothesized that integrating a disease-weighted comorbidity index into a prediction framework would improve risk stratification and facilitate targeted interventions for high-risk older adults. The resulting model may serve as a practical decision-support tool for optimizing prescribing practices and promoting safer medication use in geriatric outpatient care.

## 2. Methods

### 2.1. Study Population, Setting and Data Source

This study was conducted as part of the Hospital Prescription Analysis Cooperation Project, with data collected from six major Chinese cities: Beijing, Shanghai, Guangzhou, Shenyang, Zhengzhou, and Chengdu. A purposive sampling method was used to select secondary and tertiary medical institutions in each city. From these institutions, outpatient prescriptions for older adults were randomly sampled for 10 days each quarter (in two separate 5-consecutive-day periods). For hospitals with a Hospital Information System (HIS), electronic prescription data were extracted directly. For institutions without a complete HIS, paper prescriptions were sampled and manually entered into an EpiData database, which was then integrated into the final outpatient prescription database. During data preprocessing, duplicate prescriptions were identified based on patient IDs and ordered according to prescription date to ensure the independence of the records included in the analysis. The analysis included prescriptions for individuals aged ≥65 years seeking outpatient treatment between 1 January 2021, and 31 December 2021. Prescriptions containing only non-medicinal items, such as sterile water for injection or imaging agents, were excluded. The collected data included city, disease diagnosis, department name, drug name, dosage form, route of administration, quantity dispensed, single dose and frequency, patient gender, age, and payment method. This analysis followed the Transparent Reporting of a multivariable prediction model for Individual Prognosis Or Diagnosis (TRIPOD) publication criteria [18].

### 2.2. Diagnosis and Medication Classification

The extracted raw prescription data were managed using Microsoft Access. The data were cleaned and categorized into subgroups, with variable names including original diagnosis, year, city, and source (outpatient/inpatient). In this study, data processing was conducted for the standardized matching between the prescription diagnoses of enrolled patients and ICD-10 medical insurance diagnoses; Python 3.9.7 was adopted to construct the analytical environment. First, an ICD-10 standardized dictionary was established by integrating diagnosis names, codes and medical insurance categories, followed by text cleaning. After preprocessing the original prescription diagnoses (removing redundancies, mapping clinical abbreviations, and splitting multiple diagnoses), we adopted a hierarchical matching strategy (exact matching; fuzzy matching; semantic matching). Finally, the matching results were sorted by confidence level, and entries with a confidence level below 80% were flagged for manual review, and a standardized dataset containing patient IDs, diagnoses and matching information was generated to ensure the standardization and traceability of diagnosis information (Table S1). Medications were classified based on the Anatomical Therapeutic Chemical (ATC) classification system of the World Health Organization (WHO), using their generic names.

### 2.3. Evaluation Criteria

The Chinese criteria were applied to assess the risk of potentially inappropriate medication (PIM) use in outpatient prescriptions for older adults [13]. Eligible outpatient prescriptions were included in the assessment according to the predefined inclusion and exclusion criteria. The assessment was independently performed by two clinical pharmacists with expertise in chronic conditions using an information platform. Subsequently, a geriatric clinical pharmacist manually verified the assessment results. In cases of disagreement between the two initial assessments, consensus was reached through discussion among the three clinical pharmacists.

### 2.4. Statistical Analysis

Frequency measurements characterized categorical information, while group comparisons utilized χ^2^ analysis for categorical variables. For normally distributed continuous variables, data representation employed mean ± standard deviation (SD), whereas non-normally distributed continuous variables utilized median (interquartile range, IQR).

After adjusting for all prespecified covariates, each standardized disease indicator included in the prescriptions was individually incorporated into a multivariable logistic regression model to estimate the respective regression coefficients and *p*-values. To comprehensively characterize the collective impact of the disease spectrum within the prescriptions on the study outcome, we developed a continuous scoring variable designated as the PRCI. Diseases demonstrating statistical significance in the regression analysis were included in the index construction. Specifically, the β-coefficient for each significant disease was divided by the minimum absolute value of the regression coefficients within the group and rounded to the nearest integer. The positive or negative signs were retained to indicate the direction of the association, while diseases without statistical significance (*p* ≥ 0.05) were assigned a score of 0. The resulting PRCI for each prescription was then integrated into the prediction model as an independent risk factor.

The occurrence of PIM was defined as a binary outcome. Multivariable logistic regression was employed to identify independent predictors and construct the prediction model. The study dataset was randomly partitioned into a training set and a testing set in a 6:4 ratio. All data-driven procedures for PRCI construction, including disease selection, estimation of regression coefficients, and calculation of disease-specific weights, were performed exclusively in the training set. After the PRCI was constructed, the selected diseases and their corresponding weights were fixed and directly applied to the testing set without any re-estimation or re-selection. All statistical analyses and visualizations were performed using R software (version 4.6.0), with a predefined significance level (α) of 0.05. Odds ratios (ORs) and 95% confidence intervals (CIs) were estimated using the maximum likelihood method. A forest plot was generated based on the regression parameters to visually represent the independent effect strength of each risk factor on PIM. Furthermore, a visual nomogram was developed by converting the regression coefficients into variable-specific scoring rules. The discriminatory performance of the model was evaluated using the receiver operating characteristic (ROC) curve and the area under the curve (AUC) with its 95% CI. Calibration curves were plotted using 1000 bootstrap resamples, and decision curve analysis (DCA) was conducted to assess the clinical utility of the model. The optimal cutoff value was determined based on the principle of maximizing the Youden index (i.e., the maximum point of sensitivity + specificity − 1), and accordingly, the corresponding sensitivity (Sensitivity) and specificity (Specificity) were calculated.

## 3. Results

### 3.1. Standardized Diagnostic Mapping and ICD-10 Classification Results

A total of 131,894 raw prescription records were included in this study. The raw diagnostic text underwent rigorous preprocessing, including the removal of redundant information, the conversion of clinical abbreviations, and the decomposition of multi-diagnosis entries. Diagnostic mapping was executed within a Python 3.9.7 environment using a hierarchical strategy of exact matching, fuzzy matching (utilizing fuzzywuzzy 0.18.0), and semantic matching (utilizing text2vec-base-chinese), supplemented by a manual audit of low-confidence entries. Among the 131,894 prescription records, 4480 (3.40%) had a confidence level below 80% and were referred for manual review, while the remaining 127,414 (96.60%) had a confidence level of 80% or higher. Ultimately, 303,654 diagnostic records were identified, corresponding to 1789 standardized ICD-10 codes (Table S1).

The analysis revealed that among all standardized diagnoses, diseases of the circulatory system (ICD-10 Chapter I) accounted for the highest proportion at 33.40% (*n* = 101,430), with essential hypertension (I10.x09) and chronic ischemic heart disease (I25.103, I25.104) as the primary constituents. This was followed by endocrine, nutritional, and metabolic diseases (ICD-10 Chapter E), representing 13.53% (*n* = 41,085) of the total, where diabetes mellitus (E14) and hyperlipidemia (E78.500) were the predominant diagnoses. Diseases of the musculoskeletal system and connective tissue (ICD-10 Chapter M) and diseases of the digestive system (ICD-10 Chapter K) each accounted for 10.22% of the records (*n* = 31,048 and *n* = 31,021, respectively).

### 3.2. Basic Characteristics of Prescriptions and the Prevalence of PIMs

This study analyzed 131,894 outpatient prescriptions for older adults collected from 59 medical institutions across six cities (Beijing, Chengdu, Guangzhou, Shanghai, Shenyang, and Zhengzhou) in 2021, after excluding prescriptions for solvents, contrast agents, and other non-medicinal components. The overall prevalence of PIMs, as assessed by the Chinese criteria, was 29.00% (38,245 PIM prescriptions). Regional variations were observed, with Guangzhou exhibiting the highest PIM rate (41.72%) and Shenyang the lowest (21.60%) (Table 1).

The Chinese criteria comprise 106 relevant standards, of which 71 (66.98%) were applicable in this study. Application of the Chinese criteria identified 49,394 PIM instances within the 38,245 PIM prescriptions, with the majority (79.01%) containing only one PIM, while the proportion of prescriptions with two PIM findings was 15.17% and that with three such findings stood at 5.82%.

According to the Chinese criteria, the five most frequently detected PIMs were clopidogrel (8893, 23.25%), estazolam (6643, 17.37%), zolpidem (4510, 11.79%), insulin (4253, 11.12%), and alprazolam (3606, 9.43%).

### 3.3. Construction of the PRCI Scoring System

**Figure 1:**
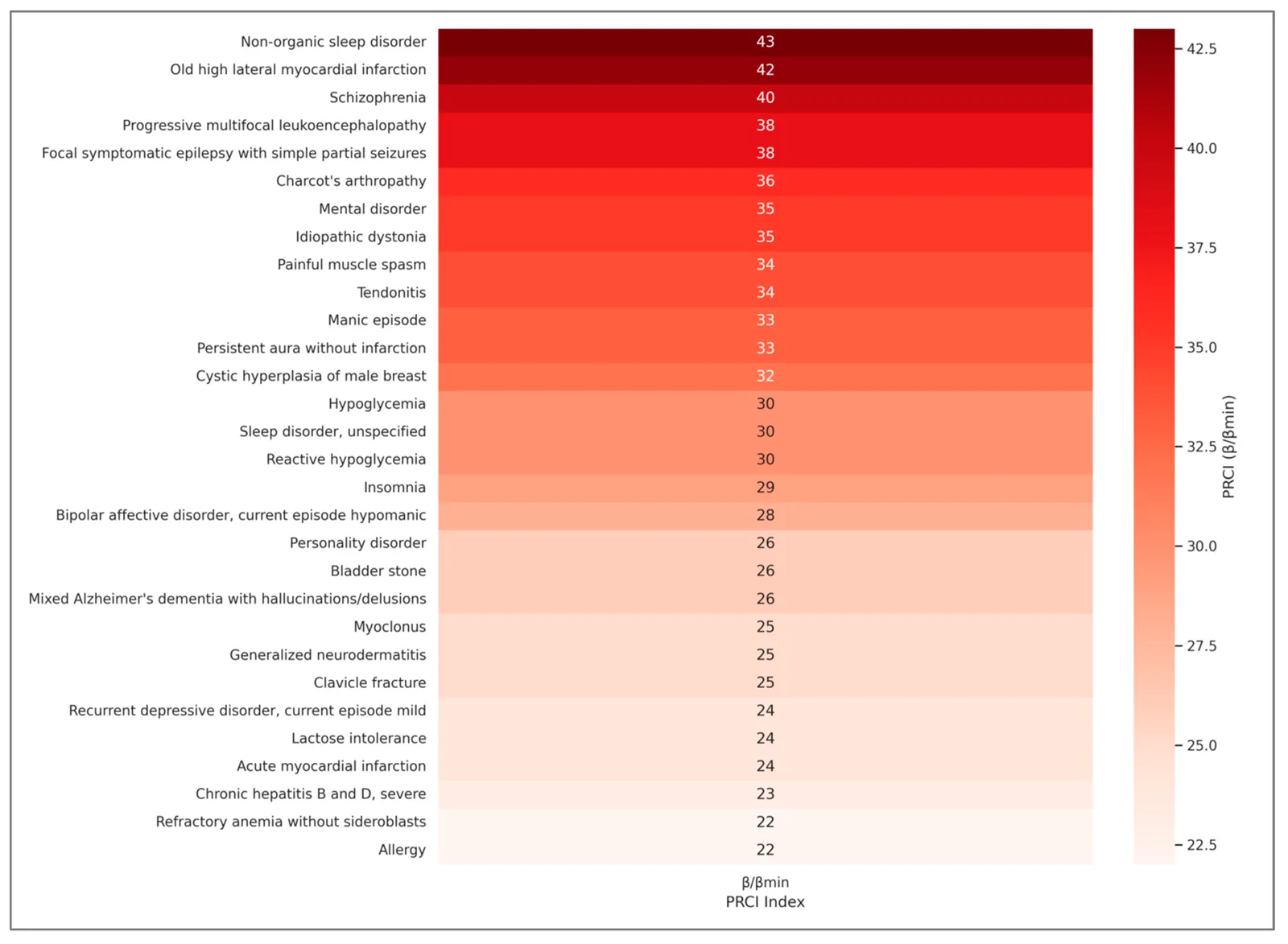
Heat map of the PIM risk-related comorbidity index (PRCI) (top 30 diseases).

The dataset was randomly divided into a training set (n = 79,136) and a testing set (*n* = 52,758) at a ratio of 6:4. In the training set, 1789 standardized disease indicators were evaluated using multivariate logistic regression. Of these, 1660 standardized diagnoses were successfully fitted, while the remaining diagnoses could not be fitted because of insufficient variation or other model-fitting limitations. Among the successfully fitted diagnoses, 252 diseases showed statistically significant associations with the study outcome (*p* < 0.05) and were therefore selected for PRCI construction (Table S2). Of these, 89 diseases were positively associated with the outcome, whereas 163 were negatively associated. For the construction of the PRCI, the absolute value of the smallest statistically significant regression coefficient was used as the reference weight, which was assigned a score of 1. The scores for the other statistically significant diseases were calculated by dividing their regression coefficients by this reference coefficient and rounding the resulting values while retaining the direction of association. Diseases without statistically significant associations were assigned a score of 0. Based on this weighting procedure, the resulting PRCI scores ranged from −41 to 43. The diseases with the highest positive PRCI scores were nonorganic sleep disorders (PRCI = 43, OR = 20.19), old high lateral myocardial infarction (PRCI = 42, OR = 18.55), and unspecified schizophrenia (PRCI = 40, OR = 15.92). In contrast, drug-induced folate deficiency anemia (PRCI = −41) and post-procedural vitreous syndrome (PRCI = −40) had the most negative PRCI scores. The top 30 standardized disease diagnoses according to PRCI score are presented in the heat map (Figure 1), with scores ranging from 22 to 43.

### 3.4. Construction of the PIM Risk Prediction Model

**Figure 2:**
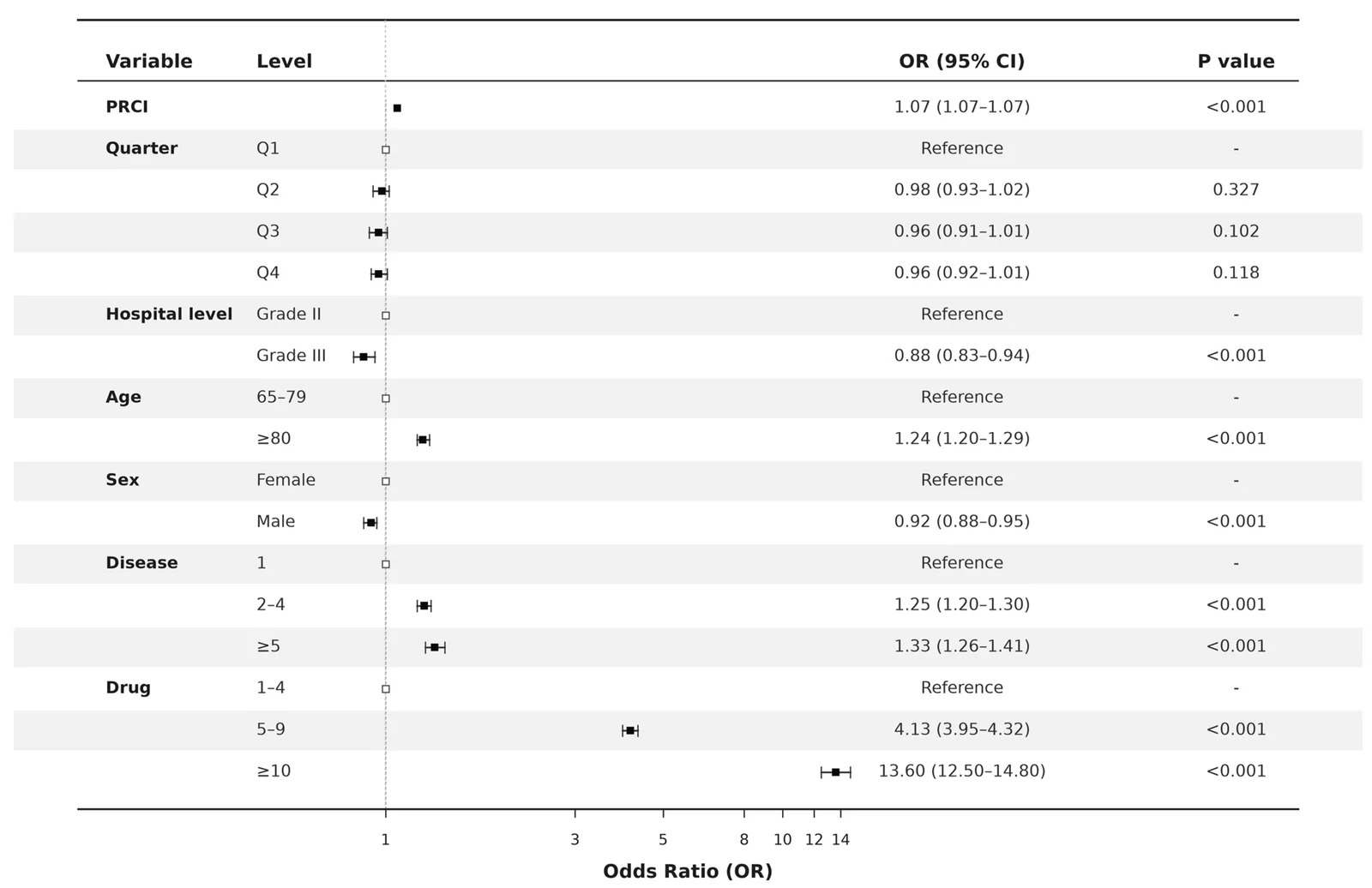
Multivariate logistic regression analysis of factors associated with PIM.Solid black squares represent the point estimates of odds ratios (ORs), horizontal lines denote the corresponding 95% confidence intervals (CIs). Open white squares indicate the reference groups. The vertical dotted line at OR = 1 is the line of no effect.

Multivariate logistic regression analysis revealed several independent predictors of PIM risk (Figure 2). Notably, polypharmacy exhibited the strongest association, with a profound dose–response relationship: compared to patients using 1–4 drugs, those prescribed 5–9 and ≥10 drugs faced a 4.13-fold (95% CI: 3.95–4.32) and 13.60-fold (95% CI: 12.50–14.80) increased risk of PIM, respectively (both *p* < 0.001). Advanced age (≥80 years; OR = 1.24, *p* < 0.001) and higher multimorbidity burden (≥5 diseases; OR = 1.33, *p* < 0.001) were also significant risk factors. For each unit increase in PRCI, the risk rose by 7% (OR = 1.07, *p* < 0.001). Conversely, being male (OR = 0.92, *p* < 0.001) and receiving care at Grade III hospitals (OR = 0.88, *p* < 0.001) were associated with a reduced likelihood of PIM. No significant seasonal variations were observed across quarters (all *p* > 0.05).

**Figure 3:**
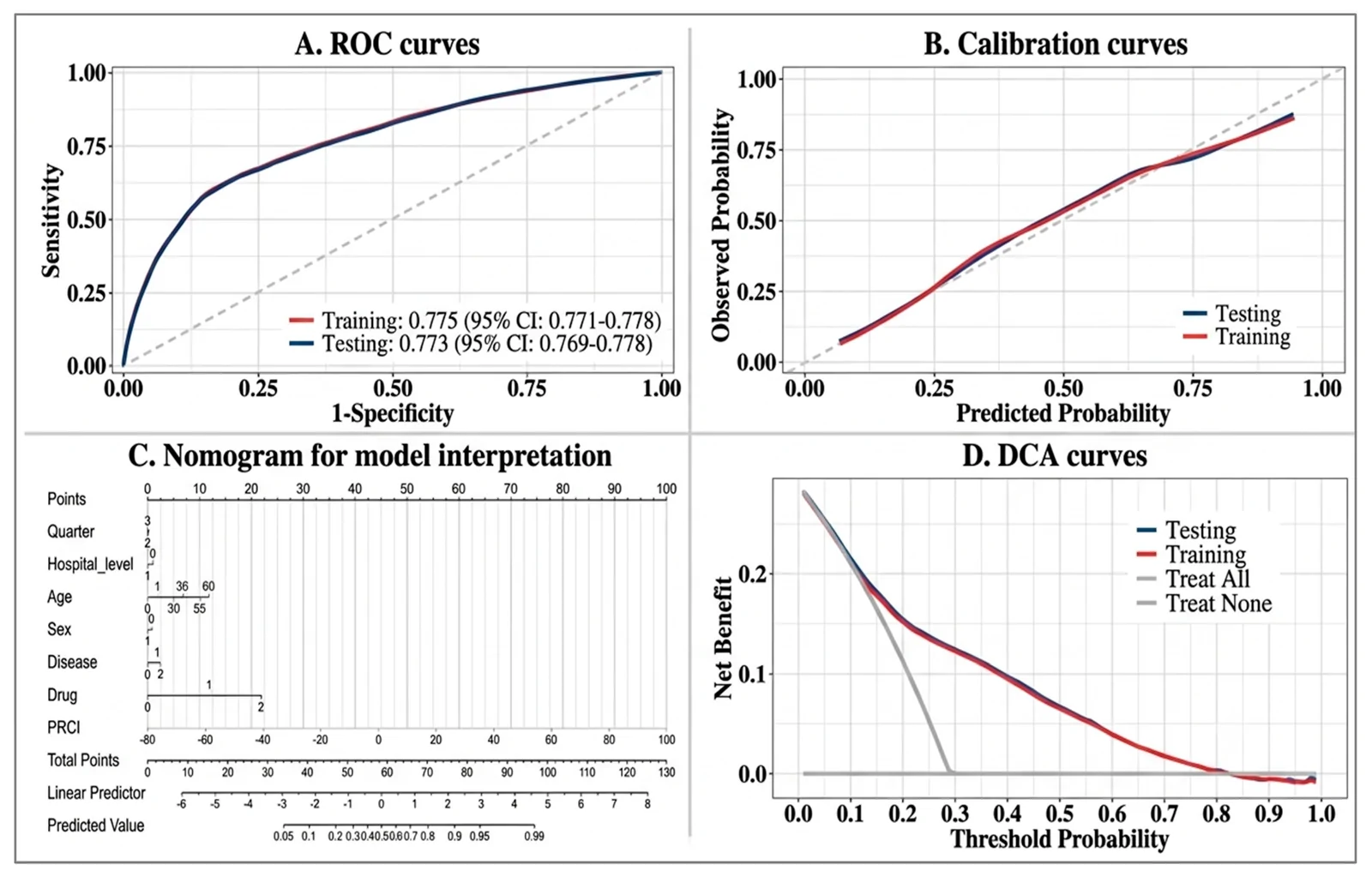
Evaluation of discrimination, calibration and clinical benefit for the risk prediction model. (**A**) Receiver operating characteristic curves in training and testing cohorts. (**B**) Calibration curves of predicted and observed probability. (**C**) Nomogram for individualized risk prediction. (**D**) Decision curve analysis to assess the clinical net benefit.

Based on the predefined variables, we constructed a PIM risk prediction model (Figure 3) and comprehensively evaluated and validated it using multiple metrics. Regarding the model’s discriminative ability (Figure 3A), ROC curve analysis revealed an Area Under the Curve (AUC) of 0.775 (95% CI: 0.771–0.778) for the training set and 0.773 (95% CI: 0.769-0.778) for the testing set. The two ROC curves largely overlapped, and the AUC values and their 95% confidence intervals remained consistent between the two datasets. For model calibration (Figure 3B), the results indicated that both the training and testing sets’ calibration curves closely adhered to the 45° ideal diagonal line. Across the entire predicted probability range from 0.0 to 1.0, the model’s risk estimates were consistent with the actual proportion of PIM occurrences. Based on the regression coefficients of each variable, this study constructed a visualized nomogram. In this nomogram (Figure 3C), a total score can be obtained by reading and summing the individual scores corresponding to the actual values of each variable. In terms of clinical utility evaluation (Figure 3D), decision curve analysis (DCA) compared the net benefit of the model’s prediction strategy with two extreme strategies: “Treat All” and “Treat None.” The results demonstrated that within a threshold probability range of approximately 0.1 to 0.8, the net benefit curves for both the training and testing sets were above the two reference lines. Within this threshold range, the clinical net benefit gained by using this model to identify high-risk patients and implement interventions was superior to that of the other two baseline strategies.

Sensitivity analyses were performed to further examine the contribution of PRCI to model performance by comparing models with different combinations of disease-related predictors (Table S3). In the training set, the AUC increased from 0.683 (95% CI: 0.678–0.687) for the Base model to 0.775 (95% CI: 0.771–0.778) after adding PRCI, while the Brier score decreased from 0.183 (95% CI: 0.182–0.184) to 0.160 (95% CI: 0.158–0.161). Similar results were observed in the testing set, with the AUC increasing from 0.682 (95% CI: 0.677–0.687) to 0.773 (95% CI: 0.769–0.778), and the Brier score decreasing from 0.184 (95% CI: 0.182–0.186) to 0.161 (95% CI: 0.159–0.163). In addition, the PRCI-only model showed higher discriminative performance than the disease-count-only model in both the training and testing sets, with AUCs of 0.677 versus 0.592 and 0.673 versus 0.595, respectively.

To assess potential multicollinearity among the predictors included in the final prediction model, variance inflation factors (VIFs) were calculated. The VIFs ranged from 1.002 to 1.214 across all predictors (Table 2). In particular, the VIFs for disease count and PRCI were 1.157 and 1.065, respectively, indicating no evidence of problematic multicollinearity among the predictors.

### 3.5. Cutoff Value and Risk Stratification Analysis for PIM Prediction

**Figure 4:**
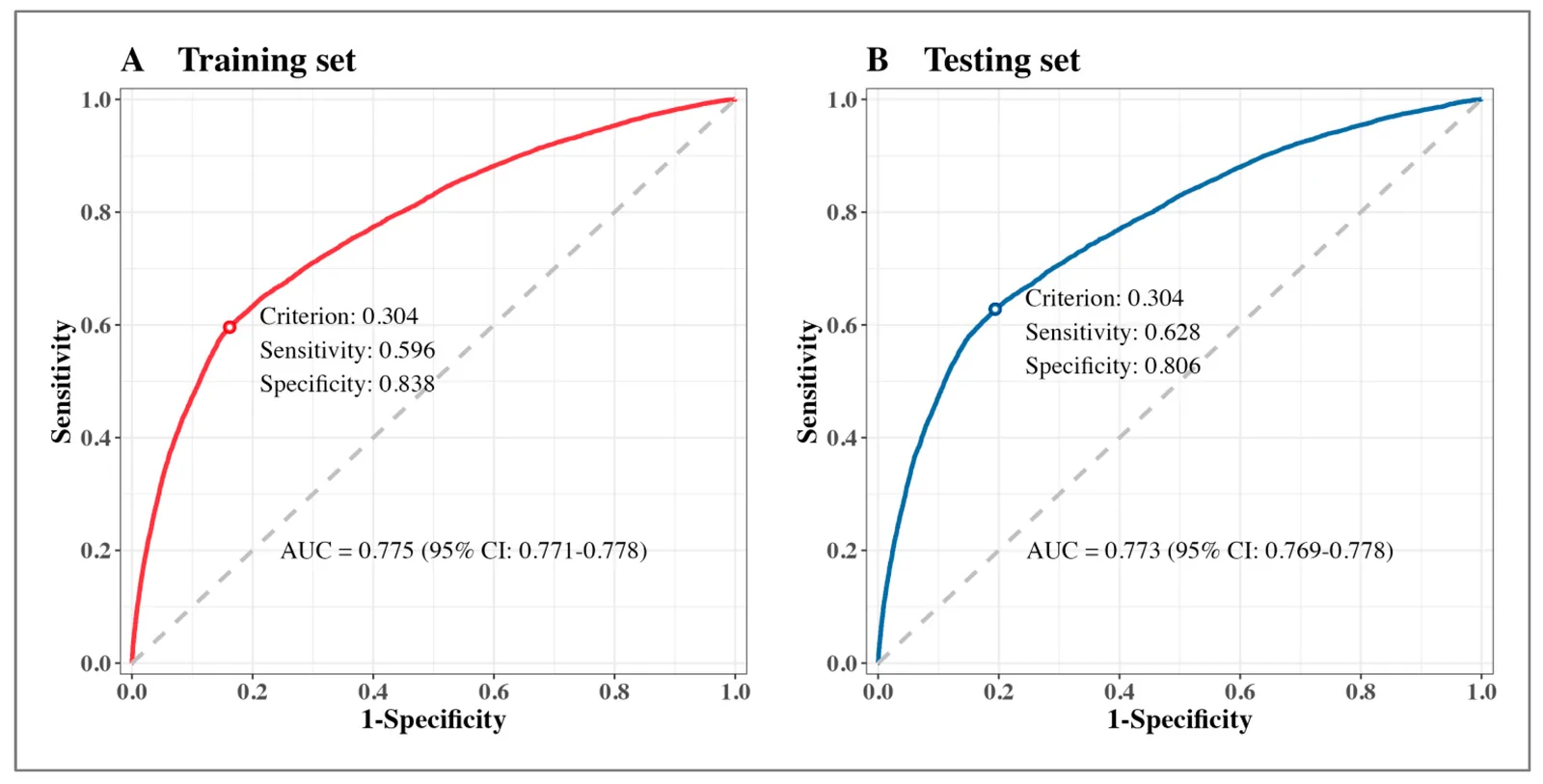
Receiver operating characteristic (ROC) curves for the PIM prediction models using the development group and validation group. (**A**): ROC curve in the training set; (**B**): ROC curve in the testing set.

The receiver operating characteristic (ROC) curve was applied to assess the discriminative performance of the prediction model for potentially inappropriate medication (PIM) (Figure 4). In the training set, the area under the ROC curve (AUC) of the model was 0.775 (95% CI: 0.771–0.778). The optimal cutoff value of the predicted probability was determined as 0.304 by maximizing the Youden index, with a corresponding sensitivity of 0.596, specificity of 0.838(Figure 4A), positive predictive value (PPV) of 0.599, and negative predictive value (NPV) of 0.837 at this threshold. The fully developed model was then applied to the testing set, which yielded an AUC of 0.773 (95% CI: 0.769–0.778). Using the same prespecified cutoff value of 0.304, the model achieved a sensitivity of 0.628, a specificity of 0.806 (Figure 4B), a PPV of 0.600, and an NPV of 0.833 in the testing set. The ROC curves and predictive performance metrics were largely consistent between the training and testing sets, indicating consistent discriminative performance within the study dataset. Risk stratification was implemented according to this cutoff: patients with a model-derived predicted probability of PIM exceeding 30.4% were classified into the PIM high-risk group, whereas those with a predicted probability of 30.4% or lower were assigned to the PIM low-risk group.

## 4. Discussion

Drawing on a large-scale, multicenter prescription database, this study developed and validated a risk prediction model for potentially inappropriate medication (PIM) in outpatient prescriptions for older adults. Based on data from 131,894 prescriptions across 59 medical institutions in six Chinese cities, we constructed the PIM Risk-Related Comorbidity Index (PRCI) and integrated it into an individualized risk prediction model for identifying prescriptions containing PIMs. The results demonstrated that the model had robust discriminative ability (AUC = 0.775), satisfactory calibration, and favorable clinical utility. Notably, the PRCI provides a novel framework for quantifying the heterogeneous contributions of different diseases to the risk of PIM-containing prescriptions, thereby enabling more precise prescription-level risk stratification than traditional multimorbidity assessment indices.

The proportion of prescriptions containing at least one PIM identified according to the Chinese criteria was 29.0%, indicating that potentially inappropriate prescribing remains an important medication safety concern in outpatient care for older adults in China. This finding is comparable to a Danish nationwide study that reported a 29% proportion of prescriptions containing at least one PIM among older adults without dementia, suggesting that potentially inappropriate prescribing remains an important challenge for medication optimization across different healthcare settings [19]. Similar proportions of PIM-containing prescriptions have also been reported in studies from other countries using explicit criteria. However, direct comparisons should be interpreted cautiously because of differences in healthcare systems, prescribing patterns, study populations, and assessment criteria [20,21].

Among all identified PIMs, clopidogrel, estazolam, zolpidem, insulin, and alprazolam were the most frequently detected medications. Notably, sedative-hypnotic agents accounted for a substantial proportion of PIMs. This observation is clinically plausible given the high prevalence of sleep disorders among older adults and the continued reliance on benzodiazepines and Z-drugs despite well-established risks of falls, fractures, cognitive impairment, and dependence [22,23]. The high frequency of insulin-related PIMs also reflects the challenges of balancing glycemic control and hypoglycemia prevention in older populations, particularly among patients with multimorbidity and functional vulnerability [24].

A major innovation of the present study is the development of the PRCI. Previous research has consistently identified multimorbidity as an important factor associated with PIM-containing prescriptions [25,26,27]. However, most studies have operationalized multimorbidity using simple disease counts or broad disease categories. Such approaches implicitly assume that all chronic conditions contribute equally to the risk of inappropriate prescribing. In reality, diseases differ substantially in their therapeutic complexity, medication requirements, monitoring demands, and susceptibility to prescribing cascades. Consequently, the contribution of individual diseases to the occurrence of PIM-containing prescriptions is unlikely to be homogeneous. To address this limitation, we developed the PRCI as a disease-weighted risk assessment tool. Rather than merely counting the number of coexisting diseases, the PRCI assigns differential weights according to the magnitude and direction of each disease’s association with PIM-containing prescriptions. The index therefore captures not only the extent of multimorbidity but also its qualitative composition. In our analysis, 252 diseases were significantly associated with PIM-containing prescriptions, of which 89 showed positive associations, and 163 showed negative associations. These findings demonstrate substantial heterogeneity in the associations between individual diseases and inappropriate prescribing and support the rationale for incorporating disease-specific weights into multimorbidity assessment.

Interestingly, several conditions with the highest PRCI scores showed clinically plausible associations with inappropriate prescribing. Nonorganic sleep disorders had the highest positive weight in the index, which may be related to the frequent use of benzodiazepines and non-benzodiazepine hypnotics in the management of sleep disorders among older adults. Likewise, psychiatric disorders such as schizophrenia often require long-term psychotropic therapy, which may increase the likelihood of prescribing medications that are potentially inappropriate for older adults. These observations suggest that the PRCI captures clinically meaningful patterns of disease-related prescribing risk rather than merely reflecting statistical associations. Furthermore, the contribution of the PRCI to the prediction model supports its potential utility as a medication-safety-oriented measure of disease-related risk in outpatient prescriptions for older adults.

Multimorbidity assessment has traditionally relied on instruments such as the Charlson Comorbidity Index (CCI) and the Elixhauser Comorbidity Index (ECI) [28,29,30]. These indices have become widely accepted tools for predicting mortality, hospitalization, healthcare utilization, and other prognostic outcomes. However, the weighting systems embedded within these instruments were developed based on outcome measures fundamentally different from medication appropriateness. The key distinction between traditional comorbidity indices and the PRCI lies in the target outcome used to derive disease weights. The CCI was originally developed to predict one-year mortality, whereas the ECI was designed to evaluate inpatient outcomes and resource utilization. Consequently, disease weights within these indices primarily reflect prognostic severity rather than prescribing complexity. Yet the mechanisms driving PIM occurrence differ substantially from those underlying mortality or hospitalization. PIM risk is more directly influenced by therapeutic burden, medication regimen complexity, disease-specific treatment strategies, and prescribing behavior. Therefore, diseases associated with high mortality risk do not necessarily confer the highest risk of inappropriate prescribing, whereas diseases requiring complex pharmacotherapy may substantially elevate PIM risk despite relatively limited prognostic impact.

Another important advantage of the PRCI is its ability to move beyond simple disease counts. Conventional multimorbidity measures generally treat all diseases as equivalent contributors to risk. Our findings clearly demonstrated that disease-specific associations with PIM varied markedly in both magnitude and direction. By incorporating these heterogeneous effects into a continuous score, the PRCI provides a more nuanced and clinically relevant characterization of medication-related vulnerability. Importantly, the PRCI is not intended to replace traditional comorbidity indices but rather to complement them. Whereas the CCI and ECI remain valuable tools for prognosis assessment, the PRCI offers a novel framework specifically tailored to medication safety and prescribing optimization in older adults.

In addition to PRCI, our findings confirmed several established predictors of PIM exposure. Polypharmacy emerged as the strongest risk factor, demonstrating a pronounced dose–response relationship. Compared with patients receiving 1–4 medications, those prescribed 5–9 medications and ≥10 medications exhibited approximately fourfold and fourteenfold increases in PIM risk, respectively. This observation is consistent with a large body of evidence identifying polypharmacy as one of the most important drivers of inappropriate prescribing [31,32,33]. As medication numbers increase, opportunities for drug–drug interactions, therapeutic duplication, prescribing cascades, and medication management errors increase substantially. Advanced age and higher multimorbidity burden also independently increased PIM risk, further emphasizing the challenges of medication management in clinically complex older populations.

The prediction model demonstrated consistent performance across multiple evaluation metrics. The AUC values were 0.775 in the training set and 0.773 in the testing set, indicating good discriminative ability according to commonly accepted standards for clinical prediction models [34]. Calibration analyses also showed good agreement between predicted and observed risks across the range of predicted probabilities. The similar discrimination and calibration performance between the training and testing sets suggests that the model maintained relatively consistent predictive performance when applied to data not used for model development. Decision curve analysis further demonstrated positive net benefit across a broad range of threshold probabilities, supporting the potential clinical utility of the model in routine outpatient medication management. Within this model, the PRCI provides an additional disease-specific dimension of risk information by incorporating the heterogeneous associations between individual diseases and PIM-containing prescriptions, complementing conventional measures of multimorbidity based on disease counts.

From a clinical perspective, the greatest value of the PRCI-based model lies in its ability to facilitate proactive rather than reactive medication safety management. Existing PIM criteria such as the Beers Criteria, STOPP/START Criteria, and Chinese Criteria are predominantly applied after prescribing decisions have already been made. In contrast, the present model enables clinicians to estimate an individual’s risk of PIM occurrence before conducting detailed medication review. This capability may allow healthcare systems to allocate pharmacist resources more efficiently by prioritizing patients most likely to benefit from medication optimization. The identified risk threshold of 30.4% further provides a practical basis for stratifying prescriptions into high-risk and low-risk groups and implementing targeted interventions.

The PRCI may be particularly suitable for integration into electronic prescribing systems and clinical decision support platforms. Because the index is derived directly from routinely collected diagnostic information, it can be automatically calculated without requiring additional clinical workload. Such integration could facilitate real-time risk alerts during outpatient prescribing and support precision medication management initiatives. Given the increasing emphasis on risk-based healthcare delivery and pharmacist-led medication review programs, disease-weighted medication safety indices such as the PRCI may represent an important step toward more individualized prescribing optimization strategies.

Several limitations of this study should be acknowledged. First, the study was conducted exclusively in a geriatric outpatient setting and did not include more complex settings, such as inpatient care, which may limit the generalizability of the findings. Second, although the model showed good internal validation performance, external validation in independent healthcare systems and diverse populations is needed to further assess its generalizability. Third, the PRCI was mainly derived from diagnostic information recorded in prescription databases and did not capture additional disease-related factors, such as disease severity or frailty, which may affect predictive performance. Thus, individual PRCI weights should be interpreted as relative contributions within the index rather than absolute disease-specific effects. Nevertheless, this approach integrates disease-specific information and heterogeneous disease–PIM associations into a practical and interpretable risk-weighting tool. Fourth, the diagnostic information used to construct the PRCI was standardized through a hierarchical mapping process, although independent validation of this process was not performed. Future studies should further evaluate its accuracy and reliability. Finally, as the model was developed using the Criteria for Potentially Inappropriate Medication for Older Adults in China, its performance may vary when other PIM assessment criteria are applied.

## 5. Conclusions

In conclusion, we developed and validated a novel disease-weighted PIM Risk-Related Comorbidity Index and incorporated it into an individualized prediction model for identifying PIM-containing outpatient prescriptions among older adults. The PRCI provides a quantitative framework for capturing the heterogeneous effects of different diseases on the risk of inappropriate prescribing and enables prescription-level risk stratification. The resulting model demonstrated favorable discrimination, calibration, and clinical utility, offering a promising decision-support tool for proactive medication safety management and precision prescribing in older adults receiving outpatient care.

## Figures and Tables

**Table 1:** Basic characteristics of older outpatients.

Characteristics	Total	Chinese Criteria		
		PIM (n)	PIM (%)	*p* Value
Total (%)	131,894	38,245	29.00	
City				<0.001
Beijing	18,429 (13.97)	6637 (17.35)	36.01	
Chengdu	8543 (6.48)	3056 (7.99)	35.77	
Guangzhou	17,737 (13.45)	7400 (19.35)	41.72	
Shanghai	44,176 (33.49)	11,084 (28.98)	25.09	
Shenyang	39,115 (29.66)	8449 (22.09)	21.60	
Zhengzhou	3894 (2.95)	1619 (4.23)	41.58	
Hospital level				0.623
Grade 2	10,314 (7.82)	3013 (7.88)	29.21	
Grade 3	121,580 (92.18)	35,232 (92.12)	28.98	
Sex, n (%)				0.081
Male	83,369 (63.21)	24,035 (62.84)	28.83	
Female	48,525 (36.79)	14,210 (37.16)	29.28	
Age, years (IQR), n (%)	80 (71, 88)			<0.001
65–79	63,893 (48.44)	16,100 (42.10)	25.20	
≥80	68,001 (51.57)	22,145 (57.90)	32.57	
No. of diseases [IQR]	1 [1,3]			<0.001
1	81,696 (61.94)	19,098 (49.94)	23.38	
2–4	33,312 (25.26)	11,455 (29.95)	34.39	
≥5	16,886 (12.80)	7692 (20.11)	45.55	
No. of medications [IQR], n (%)	2 [2,4]			<0.001
1–4	103,398 (78.39)	22,426 (58.64)	21.69	
5–9	22,008 (16.69)	11,153 (29.16)	50.68	
≥10	6488 (4.92)	4666 (12.20)	71.92	
Quarter of visit				0.016
Q1	33,656 (25.52)	9989 (26.12)	29.68	
Q2	34,041 (25.81)	9789 (25.60)	28.76	
Q3	31,190 (23.65)	8952 (23.41)	28.70	
Q4	33,007 (25.03)	9515 (24.88)	28.83	
Payment				<0.001
Free	54,134 (41.04)	14,241 (37.24)	26.31	
Partial Fee	50,893 (38.59)	14,920 (39.01)	29.32	
Full fee	26,867 (20.37)	9084 (23.75)	33.81	

PIM, potentially inappropriate medication; IQR, interquartile range; CNY, Chinese Yuan.

**Table 2:** Assessment of multicollinearity among predictors in the prediction model.

Variable	VIF
Quarter	1.002
Hospital_level	1.022
Age	1.013
Sex	1.022
Disease	1.157
Drug	1.214
PRCI	1.065

## Data Availability

The data presented in this study are available on request from the corresponding author due to privacy.

## Supplementary Materials

The following supporting information can be downloaded at: https://www.mdpi.com/article/10.3390/jcm15197675/s1.
